# Subjective Triggering Conditions of Affective Episodes in Adolescents and Young Adults from the General Population

**DOI:** 10.1192/j.eurpsy.2023.654

**Published:** 2023-07-19

**Authors:** L. Reichertz, C. Voss, K. Beesdo-Baum

**Affiliations:** Technische Universität Dresden, Dresden, Germany

## Abstract

**Introduction:**

Affective episodes often emerge in adolescence and young adulthood. Identification of factors subjectively associated with their onset may improve aetiological models and targeted intervention.

**Objectives:**

To examine precipitating conditions of (hypo-)manic and depressive episodes in adolescents and young adults from the general population.

**Methods:**

A random sample of 14-21 year-olds was drawn from the population registry of Dresden, Germany, and N=1180 were assessed in 2015/2016 (response rate: 21.7%). Lifetime depressive and (hypo-)manic symptoms as well as full-threshold depressive and (hypo-)manic episodes (DSM-5) were identified using standardized interview. Participants reporting depressive or (hypo-)manic symptoms were asked whether and which events or conditions they associate with episode onset. Besides responses on a list providing potential triggering conditions a free answer was possible. Qualitative content analysis preceded quantitative logistic regression analyses (significance level p<.05). Considered categories were: negative life events (further divided for depression into loss/danger events, burdensome life conditions, and interpersonal factors), events requiring adaptation, positive life events, internal factors, and other factors.

**Results:**

The vast majority of participants reporting depressive (n=682) respectively (hypo-)manic (n=200) symptoms also reported a precipitating condition (94.7%, 83.1%). There was no significant association between any triggering condition and the occurrence of a full-threshold depressive (n=206) or (hypo-)manic (n=25) episode. However, the number of reported categories of precipitating conditions was associated with full-threshold depressive and (hypo-)manic episodes. Among those with depressive or (hypo-)manic symptoms *and* at least one reported precipitating condition, multiple regression models including all condition categories showed that in particular internal factors, interpersonal problems and other factors were associated with the occurrence of a full-threshold depressive episode (n=199) and positive life events as well as internal factors were associated with the occurrence of a full-threshold (hypo-)manic episode (n=21).

**Conclusions:**

Adolescents and young adults from the general population usually associate the onset of phases with affective symptoms with precipitating conditions but these do not necessarily signal the emergence of a diagnostically relevant episode. Nevertheless, a greater number of and the presence of particular precipitating conditions may indicate the emergence of full-blown depressive or (hypo-)manic episodes. Thus, asking for subjective triggers appears relevant and may guide early identification and intervention.

**Disclosure of Interest:**

None Declared

